# Supplementary material for: Vat Photopolymerization 3D-Printing of Dynamic Thiol-Acrylate Photopolymers Using Bio-Derived Building Blocks
Source: Polymers (Basel). 2022 Dec 8;14(24):5377. doi: 10.3390/polym14245377 (PMC9784638; doi:10.3390/polym14245377)
Supplement: Supplementary file 1 [file polymers-14-05377-s001.zip › polymers-2081482-supplementary.pdf]

Supporting Information

Vat Photopolymerization 3D-Printing of Dynamic Thiol-Acrylate Photopolymers Using Bio-Derived Building Blocks

Usman Shaukat [1], Bernhard Sölle [1], Elisabeth Rossegger [1], Sravendra Rana [2],* and Sandra Schlögl [1],*

[1] Polymer Competence Center Leoben GmbH, Roseggerstrasse 12, 8700 Leoben, Austria; usman.shaukat@pccl.at (U.S.); bernhard.sölle@pccl.at (B.S.); elisabeth.rossegger@pccl.at (E.R.)
[2] Department of Chemistry Applied Science Cluster, School of Engineering, University of Petroleum & Energy Studies (UPES), Dehradun, 248007, India
* Correspondence: srana@ddn.upes.ac.in; Tel.: +91-9720524191 (S. R.); sandra.schloegl@pccl.at; Tel.: +43-3842-402-2354 (S. S.)

S1. Synthesis of acrylated epoxidized vegetable oil (AELO)

Acrylated epoxidized linseed oil (AELO) was synthesized by the acrylation of epoxidized linseed oil and the final product was characterized by ^{1}H NMR using $CDCl_3$ to analyze the conversion and reaction success. The signal at 2.3 ppm represents the three CH_2 groups attached at the α-position of the ester functional groups. This peak was also employed as a reference to determine the introduced acrylates (5.85, 6.15, 6.4 ppm) and unreacted epoxides (3 ppm) in the epoxidized linseed oil. The conversion of epoxide groups into acrylate groups was confirmed and the synthesized AELO was used in the formation of resin formulations.

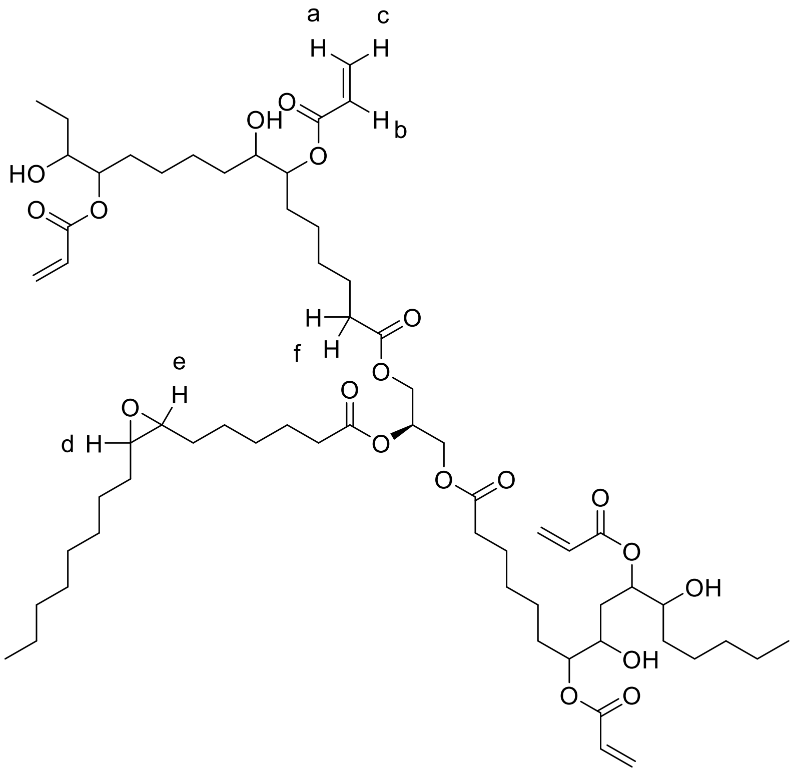

Figure S1. Structure of acrylated linseed oil (AELO).

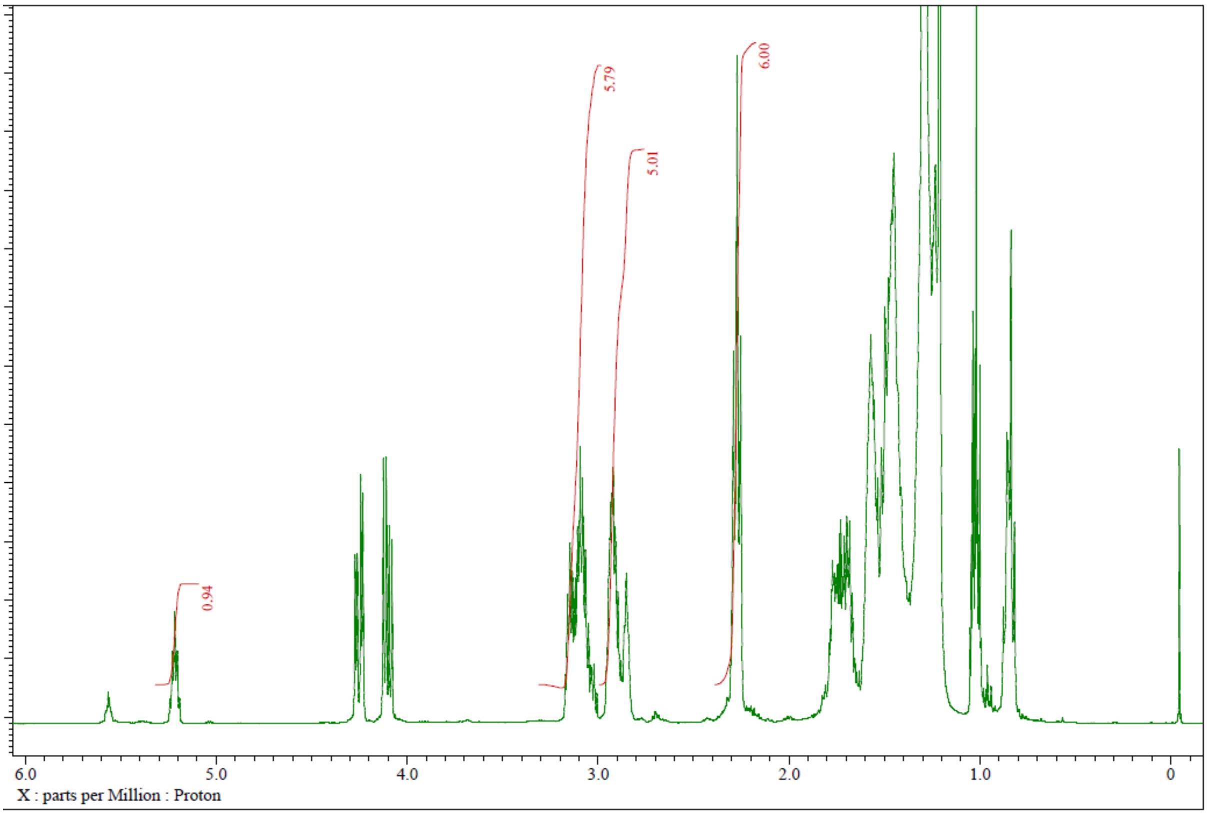

Figure S2. ^{1}H NMR spectrum of epoxidized linseed oil (ELO) in deuterated chloroform.

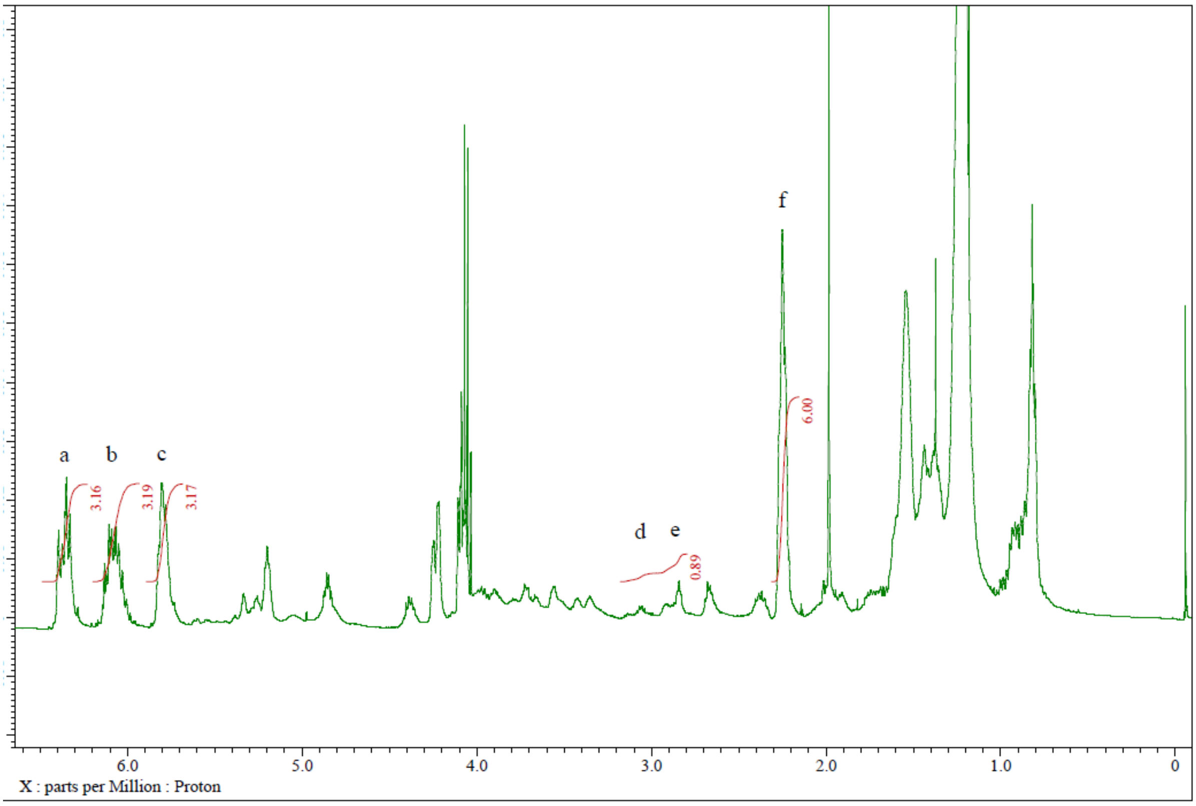

Figure S3. ^{1}H NMR spectrum of acrylated linseed oil (AELO) in deuterated chloroform.

S2. FTIR spectra

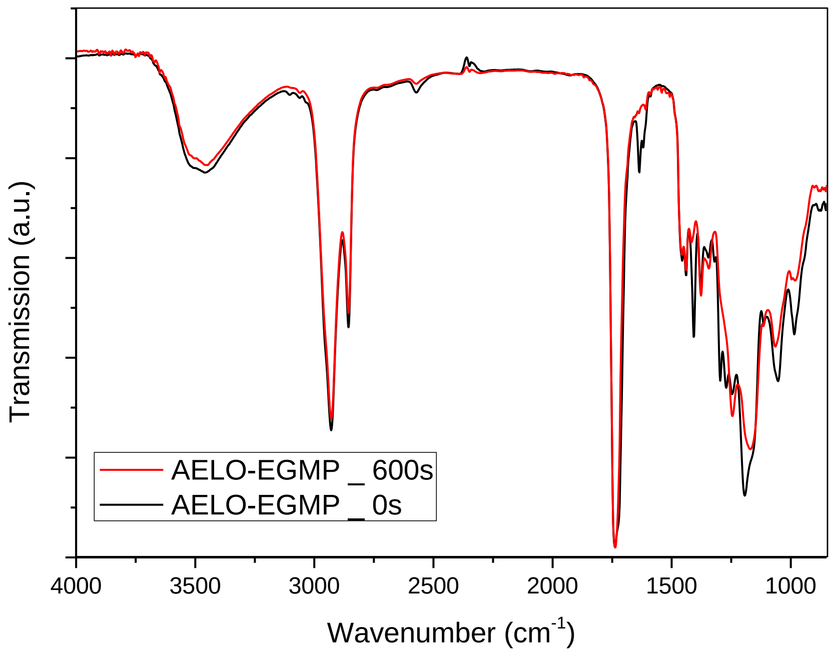

Figure S4. FTIR spectra of AELO-EGMP resin formulation prior to (0 s) and after illumination (600 s) with visible light.

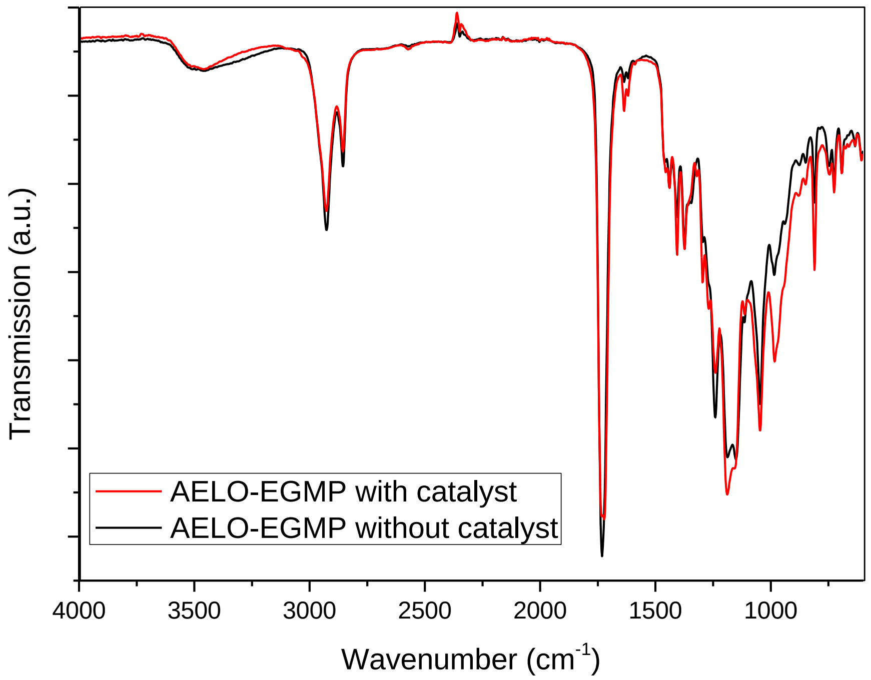

Figure S5. FTIR spectra of AELO-EGMP resin formulation with transesterification catalyst and without transesterification catalyst prior to illumination (0 s) with visible light.

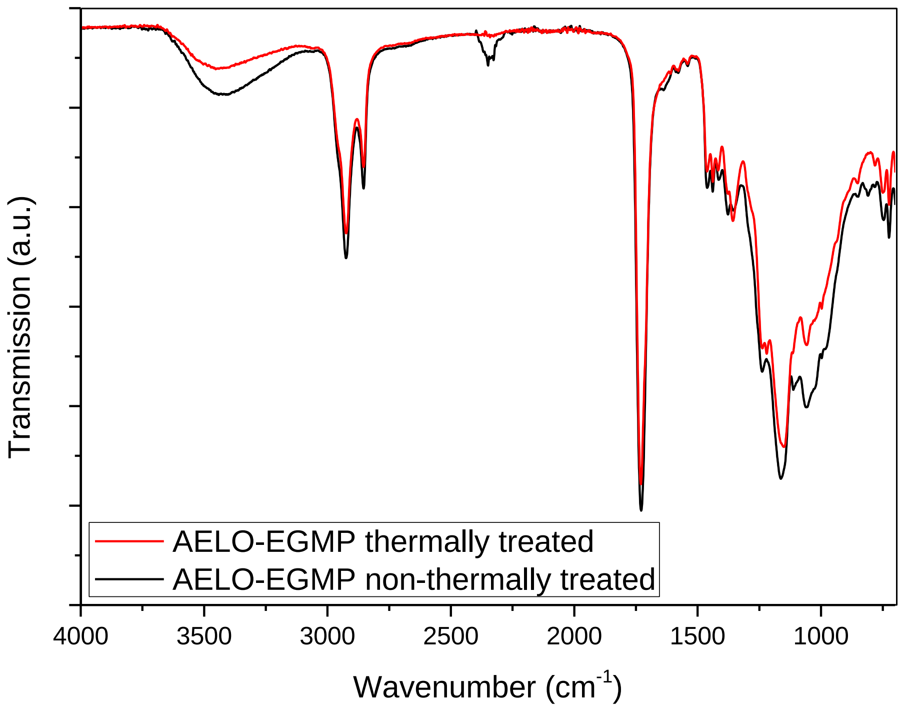

Figure S6. ATR spectra of thermally treated AELO-EGMP formulation and non-thermally treated AELO-EGMP formulation in the presence of a transesterification catalyst, after illumination (600 s) with visible light.

S3. Differential scanning calorimetry (DSC)

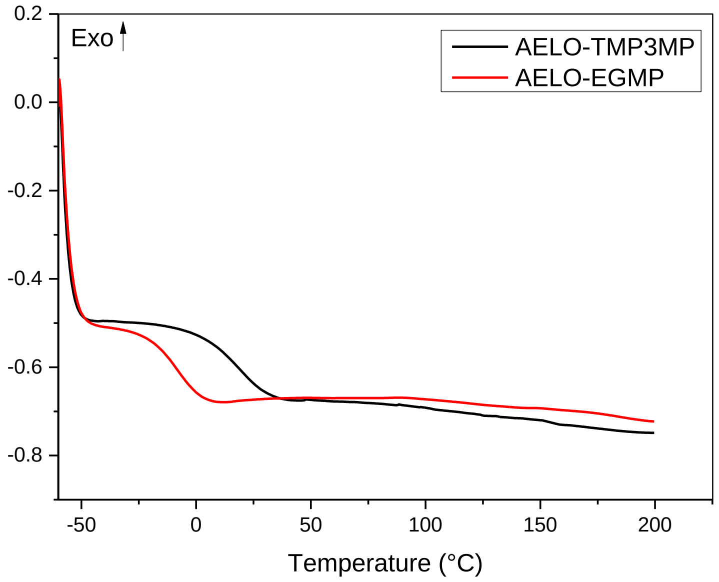

Figure S7. DSC plot of cured AELO-TMP3MP formulation and cured AELO-EGMP formulation (Midpoint T_g = –10 and –18 °C, respectively).

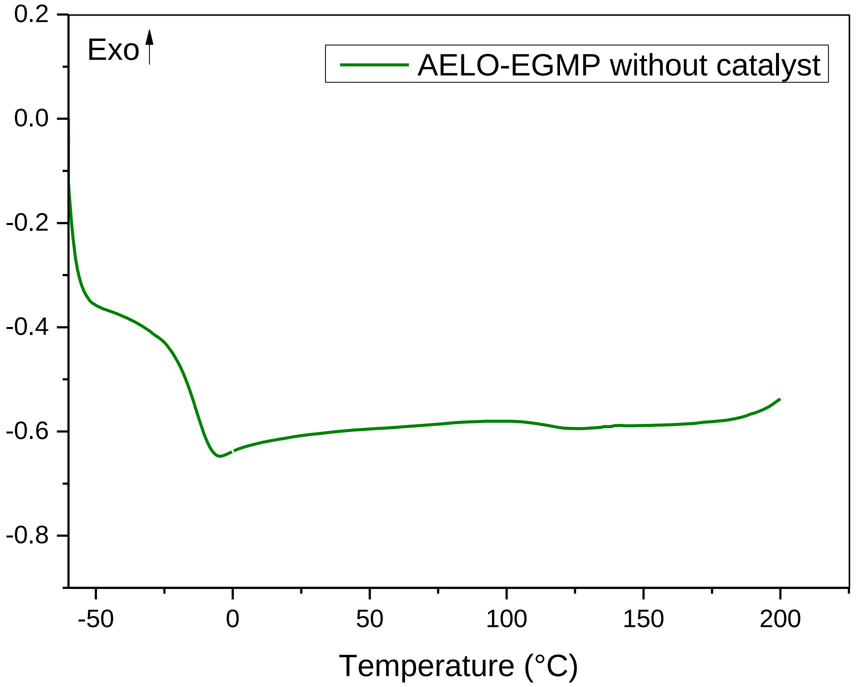

Figure S8. DSC plot of cured AELO-EGMP formulation without transesterification catalyst (Midpoint T_g = –15 °C).
